# Heart Rate Estimation Using FMCW Radar: A Two-Stage Method Evaluated for In-Vehicle Applications

**DOI:** 10.3390/biomimetics10090630

**Published:** 2025-09-17

**Authors:** Jonas Brandstetter, Eva-Maria Knoch, Frank Gauterin

**Affiliations:** 1 Faculty of Mechanical Engineering, Institute for Vehicle Systems Engineering, Karlsruhe Institute of Technology (KIT), 76131 Karlsruhe, Germany; eva-maria.knoch@kit.edu (E.-M.K.); frank.gauterin@kit.edu (F.G.); 2 Department of Complete Vehicle Data Driven Testing, Analysis and Vehicle Functions, Porsche Engineering Services GmbH, 74321 Bietigheim-Bissingen, Germany

**Keywords:** driver monitoring system, heart rate estimation, FMCW radar, vital sign monitoring, in-vehicle applications, relevance vector machine, discrete wavelet transform, Kalman filter, real-world validation

## Abstract

Assessing the driver’s state in real time is a critical challenge in modern vehicle safety systems, as human factors account for the vast majority of traffic accidents. Heart rate (HR) is a key physiological indicator of the driver’s condition, yet contactless measurements in dynamic in-vehicle environments remain difficult due to motion artifacts, vibrations, and varying operational conditions. This paper presents a novel two-stage method for HR estimation using a commercial 60 GHz frequency-modulated continuous wave (FMCW) radar sensor, specifically designed and validated for in-vehicle applications. In the first stage, coarse HR estimation is performed using the discrete wavelet transform (DWT) and autoregressive (AR) spectral analysis. The second stage refines the estimate using an inverse application of the relevance vector machine (RVM) approach, leveraging a narrowed frequency window derived from Stage 1. Final HR estimates are stabilized through sequential Kalman filtering (SKF) across time segments. The system was implemented using an Infineon BGT60TR13C radar module installed in the sun visor of a passenger vehicle. Extensive data collection was conducted during real-world driving across diverse traffic scenarios. The results demonstrate robust HR estimations with an accuracy comparable to that of commercial wearable devices, validated against a Polar H10 chest strap. This method offers several advantages over prior work, including short measurement windows (5 s), operation under varying lighting and clothing conditions, and validation in realistic driving environments. In this sense, the method contributes to the field of biomimetics by transferring the biological principles of continuous vital sign perception to technical sensorics in the automotive domain. Future work will explore the fusion of sensors with visual methods and potential extension to heart rate variability (HRV) estimations to enhance driver monitoring systems (DMSs) further.

## 1. Introduction

Assessing the driver’s state represents a central challenge in the context of modern vehicle technologies. As human error accounts for approximately 95% of all traffic accidents in the European Union (EU) [1,2], as well as around 90% in the United States (US) [3], there is a growing demand for systems capable of monitoring the driver’s condition in real time and minimizing potential risks. The increased deployment of DMSs is driven both by technological advancements and regulatory requirements. For example, EU Regulation 2019/2144 [1] mandates that vehicle manufacturers equip new vehicles with systems capable of detecting the driver’s attention, fatigue, and readiness to intervene. Moreover, the increased demand for personalized and comfort-enhancing features, both within the vehicle’s interior and in the area of advanced driver assistance systems (ADASs), has intensified the interest in DMSs further [4]. The assessment of the driver’s condition essentially involves observing the driver and deriving their vital, emotional, cognitive, and activity-related states, often based on biofeedback and vital sign data. Vital signs primarily include physiological metrics such as HR, body temperature, and respiratory rate. Biofeedback data expand this scope by incorporating additional physiological parameters, such as muscle tension and skin conductance. The overarching goal is to design DMSs deployed in real-world settings to be as non-invasive and unobtrusive as possible, in order to avoid distraction and discomfort for both the driver and passengers. Such contactless monitoring reflects a biomimetic principle, as it parallels how biological systems continuously sense and interpret subtle physiological rhythms to ensure adaptability and safety. The proposed method enables contactless HR measurements using FMCW radar.

In 25% of accidents, a temporary person-related limitation is identified as the primary cause. Within this subset, 17% are attributable to the influence of substances, 14% to fatigue, 11% to stress, and 2% to anxiety, with the remaining share linked to other causes [2]. It has been shown that alcohol and other substances increase HR [5,6,7]. HR, and particularly heart rate variability (HRV), also serve as reliable markers of states of fatigue, stress, and anxiety [8,9,10]. Monitoring HR is therefore a key parameter for assessing physiological states. In theory, this would enable up to 44% of temporary individual impairments to be detected and monitored. The challenge lies in the real-world acquisition of a driver’s HR within a vehicle, both in terms of hardware sensing and software processing. Primarily, contactless and non-invasive methods such as radar technology are employed. In addition to contactless approaches, considerable progress has also been made in contact-based methods. Different designs of steering-wheel integrated ECG electrodes have been investigated, such as printed and flexible electrodes [11], conductive-fabric-based dry electrodes [12], microneedle array electrodes and wearable EMG-based systems for steering wheel grip monitoring [13], and smart steering wheels integrating ECG, PPG, and oximetry sensors [14]. These approaches can achieve high signal quality under stable contact conditions and demonstrate the feasibility of continuous in-vehicle health monitoring. However, their performance is inherently constrained by intermittent hand contact, perspiration, and the use of gloves. In contrast, with the progression toward higher levels of automated driving (ADAS) and the increasing prevalence of hands-free functions, contact-based systems face growing disadvantages, whereas contactless radar-based sensing remains robust regardless of the driver–vehicle interaction mode.

The main difficulties on the hardware side concern the packaging and cost of the sensors. In the software domain, challenges arise from the demanding and complex signal processing required to compensate for disturbances such as motion and vibrations.

This work presents a method that was tested using a commercial off-the-shelf 60 GHz FMCW radar sensor. The method is specifically tailored to and optimized for real-world in-vehicle applications. FMCW radar is particularly well suited to this purpose, as it enables contactless measurements even through clothing. Furthermore, unlike other non-contact methods such as cameras, the technology operates independently of the lighting conditions. Privacy is also enhanced due to the non-imaging nature of the approach. The compact design and low cost of the sensors facilitate their straightforward integration into the vehicle’s interior.

Numerous approaches to determining HR and HRV based on FMCW radar signals have been proposed. The following section presents selected concepts. Broto et al. [15] evaluated a cost-effective, pulse-coherent radar sensor for contactless measurement of HR and respiration within the vehicle interior. The results demonstrate that conventional signal processing methods such as short-time Fourier transform (STFT) and empirical mode decomposition (EMD) reliably extract physiological parameters like HR and respiratory rate. Thus, the system represents a promising foundation for integration into the driver monitoring functions of modern ADASs. Singh et al. [16] investigated the use of millimeter-wave radar technology (76–81 GHz FMCW) for contactless detection of vital signs such as HR and respiration rate within vehicle cabins. In their study, various radar configurations inside the vehicle were experimentally tested. The results demonstrate that frontal placement of the radar at the level of the sternum enables reliable measurements, thus offering a promising approach for DMSs. Ni et al. [17] propose a robust method for contactless HR estimation using FMCW radar, integrating a hybrid signal model, periodic signal enhancement, and an optimization-based framework. The approach achieves high accuracy even in the presence of multilimb motion, maintaining root mean square errors (RMSEs) below 4.2 beats per minute (bpm), and does not require highly directional antennas. Yang et al. [18] propose a non-contact method for short-time HR estimation using a 60 GHz FMCW radar. They integrate improved complete ensemble empirical mode decomposition with adaptive noise (ICEEMDAN) and fast independent component analysis (ICA) to suppress respiratory harmonics and noise. The method achieves accurate HR estimations with a mean absolute error (MAE) of less than 4 bpm over 5 s intervals. Jung et al. [19] present a method for rapid remote HR measurements using a 60 GHz mmWave FMCW radar. By leveraging variations in the chirp sampling intervals within the radar’s frame structure, the authors created multiple heartbeat signals at different sampling frequencies, which were combined to improve the resolution and reduce the measurement time to just 2.5 s. The experimental results show that this approach achieves higher accuracy than that of conventional methods, even under natural breathing conditions.

The proposed method is based on a two-stage computational approach, where in the first stage, coarse estimation is performed using DWT and AR spectral analysis. Building upon this, the second stage involves a refined estimation conducted via RVM-based frequency analysis. In the final post-processing step, an SKF is applied to producing the final HR estimate. The primary focus during development was on achieving short measurement durations of only 5 s, in contrast to the significantly longer periods commonly found in the literature [20,21,22,23]. As hardware, a commercial off-the-shelf sensor was used, which was mounted onto the sun visor, considered a realistic and practical installation location. The method was exclusively validated and adapted using real-world data from actual driving scenarios to ensure realistic and reproducible accuracy. In contrast, most published methods have been evaluated under laboratory conditions. While existing studies address individual requirements such as short measurement periods, practical sensor integration, or real-world validation, none have combined all of these requirements into a single, dedicated method. The presented approach closes this gap by integrating all key requirements into one tailored solution.

## 2. Methodology

**Figure 1:**
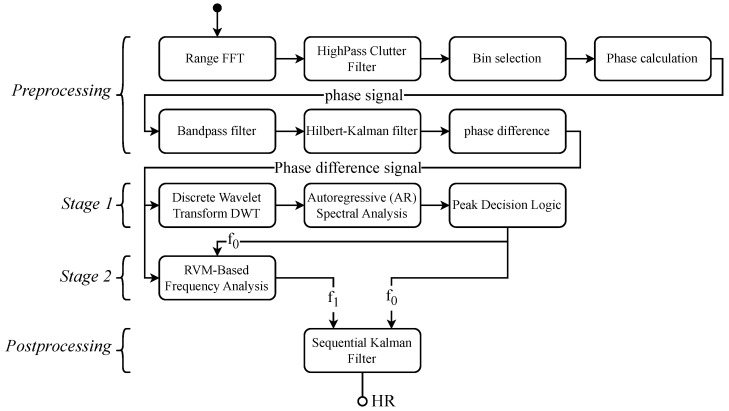
An overview of the proposed two-stage method for HR estimation using FMCW radar. The diagram highlights how the combination of coarse and fine estimation stages improves the robustness and accuracy of HR detection, representing the novel approach and central contribution of this work.

The review article by Paterniani et al. [24] clearly presents the fundamental principles. It discusses the physiological foundations, the underlying mathematical models, and the basic operating principles of radar and FMCW radar systems. The fundamental idea is to measure the movement of the human thorax caused by respiration and heartbeat using the radar Doppler effect. The typical displacement of the thorax is approximately 0.2 mm to 0.6 mm during a heartbeat and about 4 mm to 12 mm during respiration [25]. To detect these subtle movements despite all external influences and disturbances, a complex and multi-stage signal processing approach is required. Figure 1 provides an overview of the complete signal processing chain used in this study. The raw data from a radar sensor serve as the input. In this study, an Infineon XENSIV™ 60 GHz BGT60TR13C radar sensor demo board was used [26].

### 2.1. Pre-Process

In the first processing step, the input to the algorithm is the ADC samples of the received FMCW chirps at the baseband, which are resolved in range using a range fast Fourier transform (FFT), also referred to as a fast-time FFT, to localize the target object, namely the chest wall. To reduce artifacts and noise, a high-pass clutter filter (0.1 Hz) is applied to the results of the range FFT along the slow-time dimension, enabling a more precise estimation of the distance to the chest wall. These initial processing steps are found in a similar form in numerous other publications, including [17,27,28,29,30]. Subsequently, the distance at which the chest of the observed person is located is determined. Various methods exist for the selection of this bin. In this study, the distance bin exhibiting the maximum absolute amplitude is chosen. In the final step of the pre-processing, the phase signal from the selected range bin is computed. The phase ϕ(t) is calculated because minimal movements of the chest wall, caused by heartbeats or respiration, induce a time-dependent change in the propagation delay of the radar signal, leading to $\phi \left(t\right)=\frac{4\pi }{\lambda }R\left(t\right)$. Here, $\lambda =\frac{c}{f_{c}}$ denotes the wavelength of the radar, and R(t) represents the time-dependent micromovement. These changes manifest as a phase modulation of the received signal, which can be extracted through phase calculation. In a 60 GHz radar, even a micro-movement of 0.1 mm results in a phase shift of 14.4°. After the phase computation, a bandpass filter in the range of 0.5 Hz to 3.0 Hz (the typical range for HR) as well as a Hilbert–Kalman filter are applied to the data, as described by Yunlong et al. [25]. One advantage of the Hilbert–Kalman filter lies in its enhanced robustness to outliers, as large residuals are not excessively weighted. Instead of the classical update equation, a weighted update step is performed, in which the residuals are weighted differently depending on their magnitude [31]. This increases the insensitivity to measurement errors. Following the filtering step, the phase difference is computed.

### 2.2. Stage 1: Coarse Estimation

Stage 1 is used to perform an initial coarse estimation of the HR. The approximated HR serves both as the input for Stage 2 and as a basis for the final HR estimation. To mitigate potential errors in this initial estimation, the algorithm incorporates a plausibility check against the previous estimation history and applies a temporal windowing approach. Implausible jumps are suppressed, and if the Stage 1 output cannot be considered reliable, Stage 2 continues the estimation independently. This strategy further increases the robustness and stability of the overall method. First, the dominant frequencies are determined using the DWT. The DWT can be applied to non-stationary signals, which are common in biomedical signal processing. It is particularly useful because it enables a time–frequency representation [32,33].

The DWT iteratively decomposes a signal x[n] into approximation coefficients $A_{j}$ and detail coefficients $D_{j}$ by convolving it with a low-pass and a high-pass filter, respectively, followed by downsampling by a factor of two. The approximation coefficients defined in Equation (1) correspond to the low-frequency components of the signal, while the detail coefficients presented in Equation (2) represent the high-frequency components.

(1) $$\begin{array}{cc} A_{j}\left[n\right] & =\sum_{k}h\left[k-2n\right]\;A_{j-1}\left[k\right] \end{array}$$

(2) $$\begin{array}{cc} D_{j}\left[n\right] & =\sum_{k}g\left[k-2n\right]\;A_{j-1}\left[k\right] \end{array}$$

Here, h[k] denotes the coefficients of the low-pass filter (scaling function) and g[k] those of the high-pass filter (wavelet function), and $A_{0}\left[n\right]=x\left[n\right]$ represents the original input signal. The DWT decomposes the signal into multiple levels *j*, with each level corresponding to a specific frequency range. In the following, only the frequency bands A3 (0–1.25 Hz) and D3 (1.25–2.5 Hz) are considered, which correspond to an HR range of 0 to 150 bpm. The reconstruction using the inverse discrete wavelet transform (IDWT) is carried out according to Equation (3) [34].

(3) $$\begin{array}{c} A_{j-1}\left[n\right]=\sum_{k}\left(h\left[2k-n\right]\;A_{j}\left[k\right]+g\left[2k-n\right]\;D_{j}\left[k\right]\right) \end{array}$$

**Figure 2:**
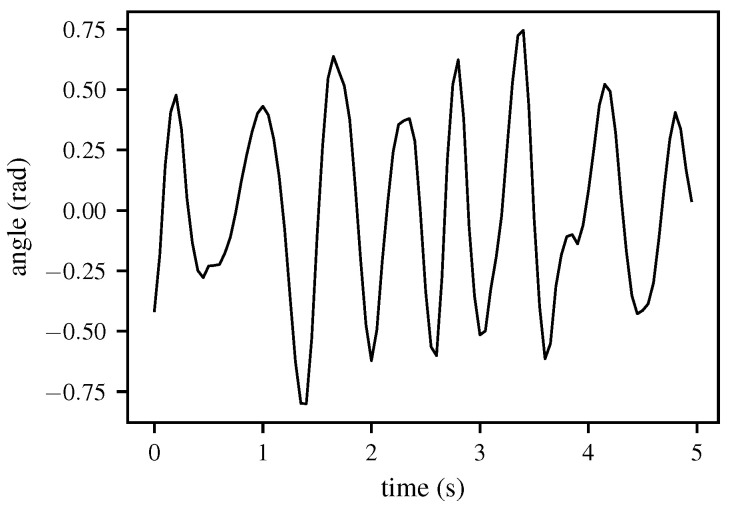
Reconstruction of the signal using the inverse DWT (IDWT), demonstrated with real data from a 5 s window. After decomposition of the radar signal with the DWT, relevant coefficients are selected and denoised before applying the IDWT. The figure illustrates that this process preserves the cardiac and respiratory components while suppressing noise.

The reconstructed signal from the inverse DWT thus consists solely of the frequency band containing the HR, as shown in Figure 2. To determine the HR from the time-domain signal, a spectral analysis is performed using an AR model, with parameter estimation carried out according to Burg’s method.

An AR model is a linear model used to describe a stationary time signal x[n] by modeling it as a weighted sum of its past values and a white noise term, as shown in Equation (4). Here, $\alpha_{k}$ denotes the AR coefficients, *p* the model order, and e[n] the white noise.

(4) $$x\left[n\right]=-\sum_{k=1}^{p}\alpha_{k}x\left[n-k\right]+e\left[n\right]$$

The objective is to determine a model of order *p* that describes the dynamic structure of the signal as accurately as possible. Such a model enables an efficient and high-resolution estimation of the Power Spectral Density (PSD), particularly for short data windows [35]. Various approaches exist for parameter estimation; in this work, the Burg method is employed [36]. The Burg method is an established technique for estimating AR coefficients. It is based on minimizing both the forward and backward prediction errors and offers, in comparison to other methods, the advantage of a high spectral resolution, particularly for short data sequences. From the estimated AR coefficients, the PSD of the signal can be computed. This is derived from the frequency response of the AR filter, as shown in Equation (5).

(5) $$P\left(f\right)=\frac{\sigma_{e}^{2}}{{\left|1+\sum_{k=1}^{p}a_{k}e^{-j2\pi fk/f_{s}}\right|}^{2}}$$

**Figure 3:**
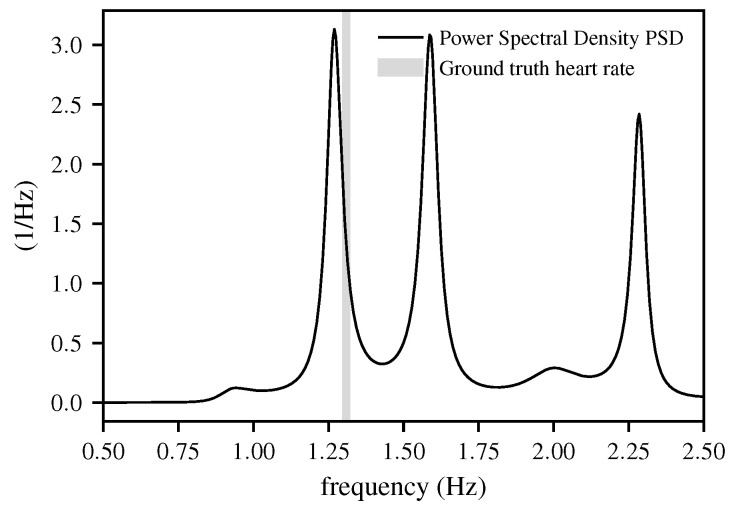
The PSD of the radar signal in the frequency band from 0.5 to 2.5 Hz, with the ground-truth HR indicated for the 5 s window displayed. The figure highlights how the spectral peak aligns with the reference HR, demonstrating that the frequency-domain representation provides a reliable basis for HR estimation.

In Equation (5), $\sigma_{e}^{2}$ denotes the variance in the white noise e[n], *f* represents the frequency axis, and $f_{s}$ is the sampling frequency. Figure 3 presents the result of the AR modeling.

Figure 3 shows that several distinct peaks can be identified, forming characteristic patterns based on their sequence and amplitude. A distinction is made between 2-, 3-, and 4-peak patterns, with the underlying logic applied to real-world data. An empirical analysis revealed that selecting solely the peak with the highest amplitude correctly identified the peak closest to the ground-truth HR in only 44.7% of cases.

**Figure 4:**
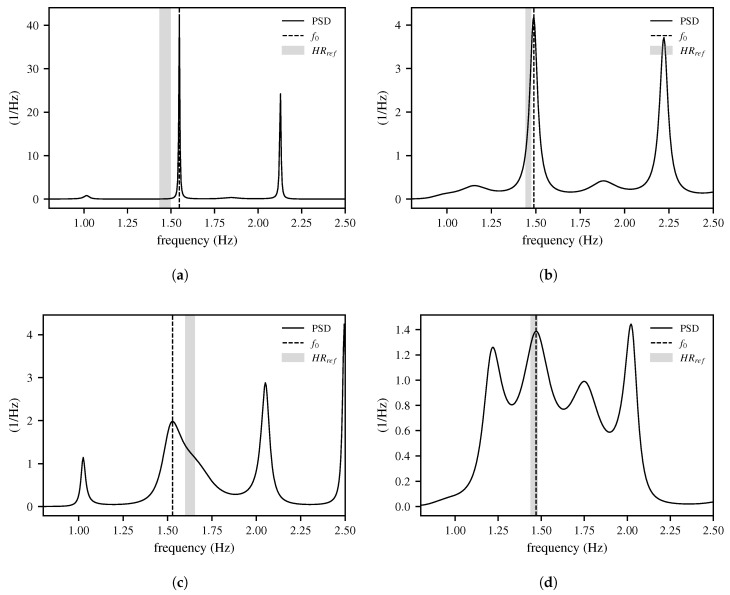
Examples of the peak decision logic, in which a peak is selected based on the peak pattern: (**a**) first peak; (**b**) first peak; (**c**) second peak; (**d**) second peak. The figure shows how varying spectral patterns can result in several candidate peaks. Analyzing the dominant peak structure within each window allows the algorithm to select the most plausible peak, which mitigates the influence of artifacts and ensures smoother HR estimation.

The corresponding results are presented in Table 1. In contrast, when the decision logic is applied, the hit rate for selecting the theoretically optimal peak increases to 80.1%. This logic makes a decision for a single peak (or the mean value between two peaks), depending on the pattern of the peaks. The frequency of the selected peak corresponds to the frequency estimate $f_{0}$ from Stage 1. Four examples of the peak decision logic are shown in Figure 4.

### 2.3. Stage 2: Fine Estimation

**Figure 5:**
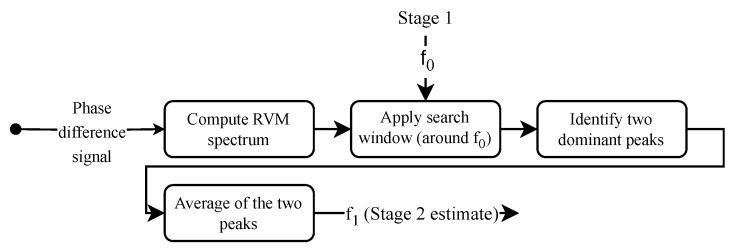
A simplified overview of Stage 2. The RVM spectrum of the phase difference signal is computed, a search window centered on the Stage 1 estimate $f_{0}$ is applied, the two dominant peaks within the window are identified, and their average defines $f_{1}$ as the refined HR estimate.

At a high level, Stage 2 can be understood as a sparse frequency selection process that refines the HR estimate provided by Stage 1. As illustrated in Figure 5, the phase difference signal is transformed into a spectrum using the inverse relevance vector machine (RVM). A search window, centered around the coarse estimate $f_{0}$ from Stage 1, constrains the frequency range of interest. Within this window, the two most dominant peaks are identified, and their average defines $f_{1}$, which serves as the refined HR estimate. The following provides a detailed description of this procedure.

Building on the Stage 1 result $f_{0}$, Stage 2 performs a refined approximation of the HR by narrowing the target range accordingly. For this purpose, an approach to solving an inverse problem is applied. The RVM approach by Tipping [37] represents a general Bayesian framework for obtaining sparse solutions to regression and classification tasks. It is a modification of the classical support vector machine (SVM), based on a probabilistic methodology, and incorporates automatic relevance determination (ARD), thereby requiring only a small number of so-called relevance vectors. The RVM can be employed as a probabilistic approach to obtaining a sparse solution for *x* in the inverse problem y=Ax+ϵ. This inverse problem can be addressed by means of sparse Bayesian estimation. In this context, the term “inverse RVM” is often used, as the RVM is applied here not for direct regression, as originally described by Tipping, but to solving inverse problems.

The RVM approach is adapted to this specific application to approximate the HR from phase difference signals. The phase difference serves as the input to the algorithm, analogous to the input used in Stage 1. The chunk (time window) over which the approximation is performed spans 5 s. At a sampling rate of 20 Hz, this corresponds to 100 values in the time vector $t={\left[t_{1},t_{2},\ldots ,t_{N}\right]}^{T}\in R^{N}$. For this time period, a frequency vector $f={\left[f_{1},f_{2},\ldots ,f_{M}\right]}^{T}\in R^{M}$ is defined, covering the range from 0.5 Hz to 2.5 Hz with 200 sampling points. Based on this frequency vector, a frequency basis is subsequently constructed as a linear combination of sine and cosine functions, as shown in Equation (6).

(6) $$\Phi_{n,m}=\left\{\begin{array}{cc} \sin(2\pi f_{m}t_{n}) & \mathrm{for}\;1\leq m\leq M,\\ \cos(2\pi f_{m-M}t_{n}) & \mathrm{for}\;M+1\leq m\leq 2M \end{array}\right.\;\mathrm{with}\;n=1,\ldots ,N.$$

In the presented method, N=100 and M=200, resulting in a frequency basis Φ with dimensions $R^{100\times 2M}=R^{100\times 400}$. Subsequently, inverse RVM regression is performed. The energy is computed from the weights of the sine and cosine basis functions $\mu \in R^{2M}$ according to (7):

(7) $$P\left(f_{m}\right)={\left(\mu_{m}^{\left(\sin\right)}\right)}^{2}+{\left(\mu_{m}^{\left(\cos\right)}\right)}^{2},\;\mathrm{for}\;m=1,\ldots ,M.$$

**Figure 6:**
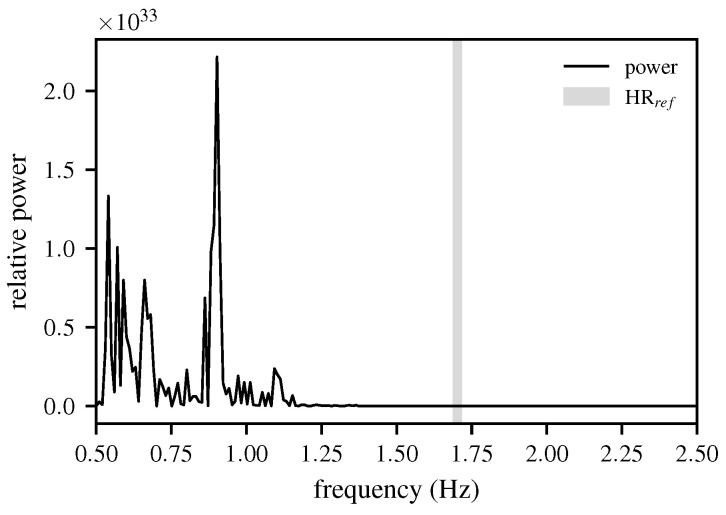
The energy spectrum based on the RVM, shown with real data from a 5 s window. The figure demonstrates that without a constrained search window, spurious peaks may dominate the spectrum, which can lead to inaccurate HR estimates.

**Figure 7:**
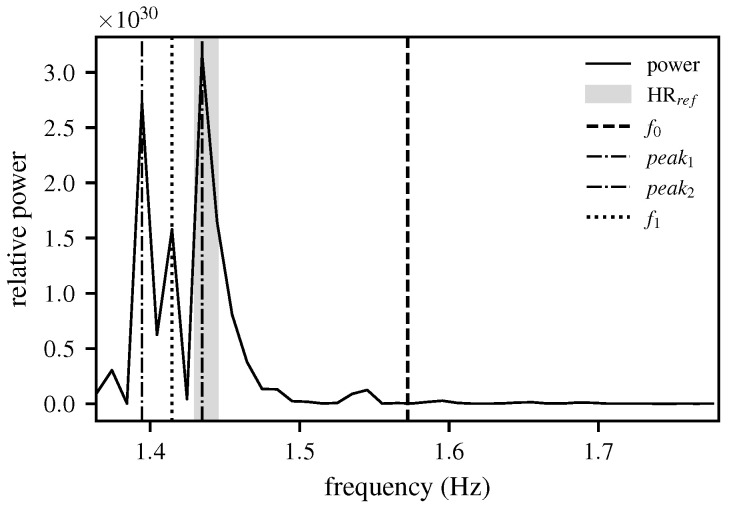
The energy spectrum based on the RVM after applying the search window. The center of the window is the result $f_{0}$ from Stage 1 ±12.5 bpm (0.208 Hz). Within the window, the mean value of the two highest peaks is used to determine $f_{1}$; shown using real data from a 5 s window. The figure highlights how constraining the search window suppresses spurious peaks and enables a more accurate HR estimation.

Such an energy spectrum is shown in Figure 6 for the selected frequency range. It becomes apparent why the search range for reliably determining the extrema must be restricted by a window and why the calculation of $f_{0}$ in Stage 1 is justified. However, if the search window is restricted to a range of ±12.5 bpm around the target HR $HR_{ref}$, as illustrated in Figure 7, significantly more precise peak detection can be performed. The detection of local extrema is carried out across the entire search space. As a result, the mean value of the two peaks with the highest amplitude within the considered range is used. The special case where no peak is detected within the search space is addressed by determining the frequency at which the mean energy within the window is closest to the target energy value. The result of Stage 2 is the approximated HR $f_{1}$.

### 2.4. Post-Processing

As a result of Stage 1 and Stage 2, the approximated HR $f_{0}$ and $f_{1}$ values are obtained. In the post-processing step, these values are filtered across multiple chunks, i.e., over a longer time period. Consequently, the problem can be defined as a sensor fusion task involving two sensors. To solve this problem, an SKF is employed. In this approach, the Kalman filter uses two independent sensor signals for state estimation. Here, the state estimation is based on two independent measurement signals, $f_{0}\left(t\right)$ and $f_{1}\left(t\right)$, which observe the same system state. Instead of employing an augmented state space, the two measurements are processed sequentially within the standard Kalman filter update. This enables simple and computationally efficient sensor fusion without modifying the underlying state model. The method is particularly well suited to systems with multiple noisy measurement channels [38,39]. The dynamic system is described by the state vector $x_{k}\in R^{n}$, as well as by the two scalar quantities $f_{0}\left(k\right)$ and $f_{1}\left(k\right)$, which refer to the same state as that in Equation (8). Here, $v_{i}\left(k\right)$ denotes the measurement noise.

(8) $$f_{i}\left(k\right)=Hx_{k}+v_{i}\left(k\right),\;i=0,1$$

The SFK algorithm consists of two main steps: the prediction according to Equation (9) and the sequential updates, as presented in Equation (10).

(9) $$\begin{array}{cc} {\hat{x}}_{k|k-1} & =A{\hat{x}}_{k-1|k-1} \end{array}$$

(10) $$\begin{array}{cc} P_{k|k-1} & =AP_{k-1|k-1}A^{⊤}+Q \end{array}$$

**Figure 8:**
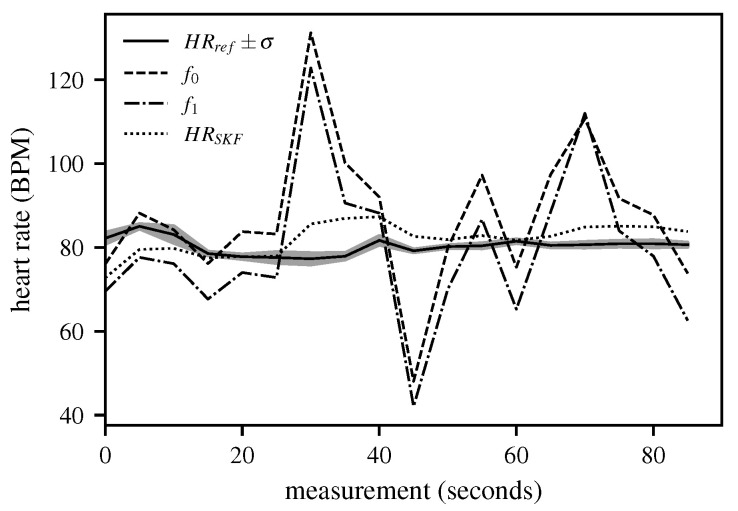
Representative results after applying the sequential Kalman filter over 90 s (corresponding to 18 5-s chunks). It shows the ground-truth measurement $HR_{\mathrm{ref}}$ over the entire period, as well as the results from Stage 1 ($f_{0}$), Stage 2 ($f_{1}$), and after post-processing ($HR_{\mathrm{SKF}}$). The comparison illustrates how post-processing with the Kalman filter smooths fluctuations from earlier stages and provides the final stable HR estimate.

In this context, ${\hat{x}}_{k|k-1}$ denotes the a priori estimate prior to the measurement, and ${\hat{x}}_{k-1|k-1}$ represents the a posteriori estimate after incorporating all measurement data. The matrix *A* is the system matrix, *P* is the covariance matrix, and *Q* is the process noise covariance matrix. In the subsequent update step, the objective is to improve the a priori estimate ${\hat{x}}_{k|k-1}$ and its associated uncertainty $P_{k|k-1}$ by incorporating the current measurements. In the SKF, this step is performed sequentially, first using $f_{0}\left(k\right)$ and then $f_{1}\left(k\right)$. For the update with $f_{0}\left(k\right)$, the Kalman gain is first computed as $K_{0}=P_{k|k-1}H^{⊤}{\left(HP_{k|k-1}H^{⊤}+R_{0}\right)}^{-1}$, followed by the state correction ${\hat{x}}_{k}^{\left(0\right)}={\hat{x}}_{k|k-1}+K_{0}\cdot \left(f_{0}\left(k\right)-H{\hat{x}}_{k|k-1}\right)$, and finally, the covariance correction is performed as $P_{k}^{\left(0\right)}=\left(I-K_{0}H\right)P_{k|k-1}$. Analogously, the second update step is performed, now starting from the intermediate solution ${\hat{x}}_{k}^{\left(0\right)},P_{k}^{\left(0\right)}$, for the update with $f_{1}\left(k\right)$. First, the Kalman gain is computed as $K_{1}=P_{k}^{\left(0\right)}H^{⊤}{\left(HP_{k}^{\left(0\right)}H^{⊤}+R_{1}\right)}^{-1}$. Next, the state correction is performed, ${\hat{x}}_{k|k}={\hat{x}}_{k}^{\left(0\right)}+K_{1}\cdot \left(f_{1}\left(k\right)-H{\hat{x}}_{k}^{\left(0\right)}\right)$, and finally, the covariance correction is carried out: $P_{k|k}=\left(I-K_{1}H\right)P_{k}^{(}0)$. Figure 8 shows the results for a measurement file with a duration of 90 s.

## 3. Results

For the acquisition of radar data, an Infineon XENSIV™ 60 GHz BGT60TR13C radar sensor demo board was used [26]. This board was operated directly in a vehicle in combination with a Raspberry Pi 5 [40]. The configuration of the radar board is provided in Table 2. Ground-truth HR data were acquired using a Polar H10 chest strap [41].

The radar sensor was installed in the sun visor. The distance between the sensor and the chest was approximately 50 cm, at an angle of 60° to the chest. The data were recorded during regular driving operation and covered a wide range of different scenarios, including highway driving, urban traffic, and traffic congestion, both with and without a front passenger. The driver’s behavior was not influenced in any way, and he behaved as he would during normal driving. Each recording was conducted over a period of 90 s, with the intervals between individual recordings varying randomly.

In total, 74 measurement sessions were conducted using a single adult male subject with a normal body constitution. While the dataset is thus limited to one participant, multiple variations were systematically included to evaluate the robustness of the method under realistic conditions. The recordings covered a total duration of 111 min, corresponding to 1332 five-second chunks. The driving scenarios comprised highway driving, urban traffic, and stop-and-go congestion, as well as driving on road segments with both smooth and rough surfaces. Measurements were performed both with and without a front passenger present. In the sessions with a passenger, natural interactions such as conversation took place. Importantly, the driver’s behavior was not influenced in any way; no consideration was given to the ongoing measurements, and driving as well as in-vehicle activities were performed as under normal everyday conditions. The rear seats were unoccupied at all times. The radar was mounted onto the sun visor and aligned such that its beam physically covered only the thorax region of the driver, ensuring that no radar reflections from the passenger could influence the results. To increase the variability further, different clothing conditions were tested. These variations ensured that the dataset captured a representative range of environmental and operational factors typically encountered in everyday driving, even though subject diversity in terms of sex, age, and body constitution was not addressed in this proof-of-concept study.

The results of the proposed method for this dataset are presented in Table 3. Across the entire dataset, an overall relative error of 6.88% was obtained. In addition, the results further classify errors as out-of-1-SD and out-of-2-SD cases. An error is recorded only if the deviation from the ground-truth measurement exceeds one or two standard deviations (1·SD or 2·SD) during the measurement period.

**Figure 9:**
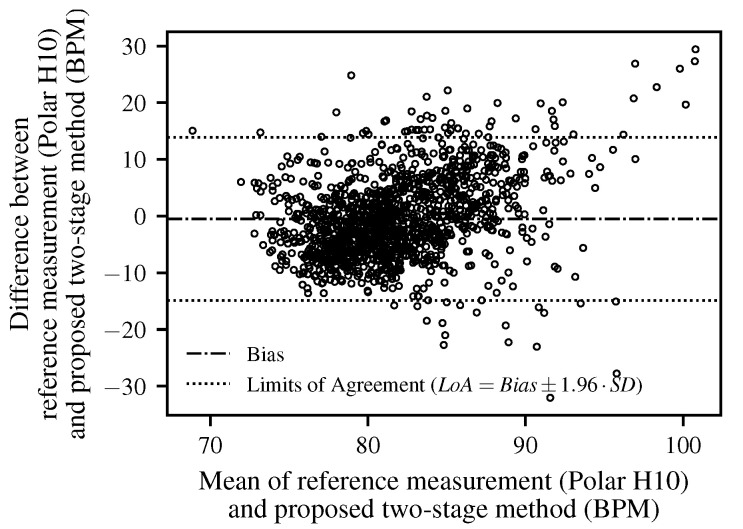
A Bland–Altman plot comparing the HR estimated using the proposed two-stage method with the Polar H10 reference. Each dot represents an individual paired measurement (difference versus mean). The solid line indicates the mean bias, while the dotted lines represent the 95% limits of agreement (LoA=Bias±1.96·SD).

In addition to the MAE and MRE values, a Bland–Altman analysis was performed to evaluate the agreement between the proposed method and the Polar H10 reference device. Figure 9 shows the differences plotted against the mean of the two measurements. The mean bias was −0.48 bpm, and the 95% limits of agreement (LoA=Bias±1.96·SD) ranged from −14.9 bpm to 13.9 bpm. The majority of data points fall within these limits, indicating good agreement. The majority of measurements fell within these limits.

**Figure 10:**
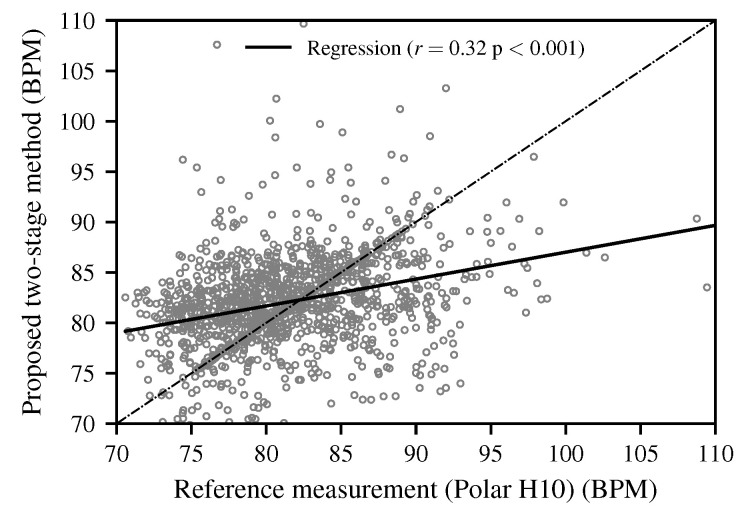
A scatter plot of the HR estimates obtained with the proposed two-stage method versus the Polar H10 reference. Each dot represents an individual paired measurement. The dashed line indicates the line of identity (y=x), while the solid line shows the linear regression fit (r=0.32,p<0.001).

In addition to the Bland–Altman analysis, a scatter plot of the estimated HR versus the reference values (Figure 10) was generated. The analysis yielded a Pearson’s correlation coefficient of r=0.32 (p<0.001), indicating a weak but statistically significant linear association between the two methods.

## 4. Discussion

As shown in the results, the error after post-processing amounts to a 6.9% mean relative error (MRE). To put this value into perspective, reference values from the literature for wrist wearables are provided. Shcherbina et al. [42] report an MRE between 1.8% and 5.5%. In the study by Etiwy et al. [43], the MRE ranges from 4.1% to 13%. Germini et al. [44] determined error values between 2.4% and 17% for common wearables. Comparisons between the results of the proposed method and prior work utilizing FMCW radar for in-vehicle HR estimation reveal varying levels of performance. In the work by Castro et al. [45], a MAE of 3.51 bpm overall, 5.44 bpm for highway driving, and 1.97 bpm for urban driving was reported. In the study by Broto et al. [15], the MRE ranges between 10.65% and 14.77%. The Polar H10 chest strap used in this study is recognized in the literature as a precise reference standard and achieves an accuracy of less than 1% MRE [43,46,47,48].

Beyond these comparisons, the present study contributes several aspects that distinguish it from prior work. First, the proposed method achieves a robust performance using short measurement windows of only 5 s, whereas many published approaches rely on substantially longer durations. Second, validation was conducted exclusively under real-world driving conditions rather than in controlled laboratory environments, ensuring that the reported accuracy reflected realistic operational scenarios. Third, the sensor was mounted in the sun visor, a practical and production-feasible installation location. By combining these requirements within a single framework, the proposed method not only achieves competitive error values but also demonstrates practical feasibility for integration into driver monitoring systems.

The Bland–Altman analysis confirmed the overall agreement between the proposed method and the reference device. While most values were within the 95% limits of agreement, a slight increase in variance was observed at a higher HR. This trend is consistent with previous radar-based HR estimation studies. For instance, Huang et al. demonstrated real-time HR detection using a 77 GHz FMCW radar and reported a reliable performance but also highlighted that overlapping respiratory harmonics and an elevated HR can lead to reduced accuracy [49]. Similarly, Xu et al. developed a higher-order harmonic peak selection method for radar-based monitoring and noted that estimation errors increase when cardiac frequencies approach respiration-related components, particularly at higher physiological rates [50]. These findings underline that additional measurements covering higher HR ranges are required to draw more conclusive insights and to comprehensively evaluate the robustness of the proposed method. The correlation analysis further revealed only a weak but statistically significant linear association between the proposed method and the Polar H10 reference (r=0.32,p<0.001). Although this value appears low, it is consistent with the intended application domain and reflects the known challenges of radar-based HR estimation in real-world in-vehicle settings, particularly due to motion artifacts and overlapping respiratory harmonics. For driver monitoring, the primary objective is not an exact reproduction of the absolute HR values but the reliable detection of changes and trends in the driver’s physiological state. In this respect, the proposed method provides sufficient sensitivity to variations in HR across diverse driving conditions, which is adequate for enhancing driver monitoring systems rather than for clinical diagnostics. Beyond these findings, it is important to explicitly acknowledge several limitations of this work and to outline directions for future research.

While the proposed method demonstrated promising results, several limitations need to be acknowledged. The dataset was limited to a single subject. Although multiple driving scenarios and road conditions, clothing variations, and the presence of a passenger were included, subject diversity in terms of age, sex, and body constitution was not addressed. Future work should therefore extend validation to larger and more diverse cohorts, including participants with rhythm disturbances such as arrhythmia. External conditions also strongly affect radar-based HR estimations. The radar sensor was mounted in the sun visor, which represents a realistic installation scenario for series production. In this configuration, the radar beams strike the thorax at an angle of 60°. Contrary to previous assumptions, Ahmed et al. [51] demonstrated that at short distances (<0.9 m), the MAE at incidence angles of 20° and 40° is not significantly worse than that at an angle of 0°. Therefore, the chosen installation configuration does not negatively impact the results. In contrast, the clothing worn has a considerably greater influence. Depending on its thickness and the material properties, clothing attenuates and scatters the radar signal. Additionally, mechanical decoupling occurs, meaning that the clothing moves differently than the thorax, which can be caused by vehicle vibrations, body movements, or respiration. These movements superimpose onto and complicate precise HR detection. A typical respiratory rate in the range of 0.1–0.5 Hz overlaps, through its second harmonic, with the HR range of 0.8–2.5 Hz, which complicates frequency separation further. In the transmission of vibrations from the vehicle to the driver, very low-frequency vibrations in the range of 0.5–5 Hz are particularly disruptive, mainly caused by road surface irregularities and leading to vertical body movements [52]. Together, these factors represent the main external influences that degrade accuracy. Future work should therefore explore robustness enhancements, for example, through adaptive filtering, sensor fusion, or integration with complementary modalities such as camera-based remote photoplethysmography. Moreover, the study design focused on single-person monitoring. Although sessions with a front passenger were included, the radar was physically aligned to cover only the driver’s thorax, and the rear seats were unoccupied. Multi-person interference was therefore deliberately excluded to ensure a controlled evaluation. Nevertheless, this represents an important limitation that warrants systematic investigation in future work. In addition, the computational cost and feasibility of real-time deployment were considered. The data acquisition was performed with a Raspberry Pi 5, while all computations were carried out on a MacBook M2 [53]. The achieved processing times confirm that the pipeline is sufficiently lightweight to allow for real-time operation in production vehicles. The algorithm can therefore be distinguished from the deep-learning-based approaches reported in the literature, which have often required dedicated Graphics Processing Units (GPUs) and higher resource budgets. In contrast, the present pipeline is modest in its computational demands and thus well suited to deployment on embedded platforms. This is particularly relevant as the trend toward software-defined vehicles (SDVs) is accompanied by the introduction of powerful central computing platforms hosting high-demand functions such as ADASs and Highly Automated Driving (HAD). Against this background, the low computational cost of the proposed method represents a clear advantage for integration. Moreover, due to the modular and sequential structure of the algorithm, individual software modules can be updated, optimized, or replaced independently. This enables continuous improvement and extension of its functionality, including in series production via over-the-air (OTA) updates, thereby ensuring long-term adaptability and maintainability. Finally, the choice of the Polar H10 chest strap as a reference was based on its proven accuracy and practicality in real-world driving. While ECG would provide the clinical gold standard, the contactless approach investigated here is not intended to reach medical-grade accuracy, and an ultra-precise reference would not necessarily provide additional benefit for the intended driver monitoring application. Beyond the potential of extending the method toward HRV estimation and sensor fusion with visual modalities, several challenges must be addressed to realize these prospects. First, synchronization between radar and camera signals is non-trivial, as different sampling rates and possible latency can compromise the temporal alignment required for robust multimodal fusion. Second, the integration of multiple sensing modalities inevitably increases the algorithmic complexity and computational demand. While the present pipeline is lightweight and suitable for embedded platforms, multimodal fusion may require more advanced hardware or optimized software architectures. Third, sensor fusion introduces challenges related to data quality and reliability since each modality is subject to specific disturbances (e.g., lighting conditions for visual data, vibrations for radar), and strategies for handling contradictory or missing signals must be developed. Finally, for HRV estimation in particular, longer and artifact-free recordings are essential, as HRV metrics are highly sensitive to noise and missing beats. Addressing these aspects is crucial to establishing a realistic and scalable roadmap for future driver monitoring systems.

## 5. Conclusions

The accuracy of the method presented here is within the range of typical wrist wearable devices, as well as that reported in publications utilizing FMCW radar for HR approximation. It should be noted that in the context of in-vehicle FMCW radar applications, publications do not always clearly indicate how realistic the test data used actually are, nor how the performance of the methods was determined based on these data. However, the origin and quality of the data used have a substantial impact on the performance of the method. Under laboratory conditions, significantly better results can be achieved compared to those obtained with real-world application data. Use in vehicles, where HR is employed as an information source for DMSs, likewise does not require the same level of accuracy as a medical device. Rather, the focus is on changes and trends.

In addition, the Bland–Altman analysis confirmed the overall agreement between the proposed method and the chest strap reference. The mean bias and narrow limits of agreement underline the validity of the approach across diverse driving conditions. The correlation analysis likewise indicated a statistically significant, though weak, linear association with the reference. However, a slight increase in variability was observed at a higher HR, which indicates the need for additional measurements under such conditions to strengthen the evaluation.

Future work should first investigate and clarify the patterns underlying the peak decision process and explain this behavior more comprehensively. While the results are promising, it should be noted that the present evaluation was based on a single subject and did not include cases of arrhythmia or multi-person scenarios, which limits the generalizability of the findings. Furthermore, the method can be combined with visual data. On the one hand, sensor fusion can be implemented to detect movements. On the other hand, an additional approximation of the HR can be achieved using remote photoplethysmography (rPPG) methods. It should also be examined to what extent the proposed method can be extended to approximate HRV data. These data carry high informational value and can significantly enhance DMS applications.

## Figures and Tables

**Table 1:** This table shows the improvement achieved by employing a peak decision logic that selects the peak based on a defined decision logic, rather than simply choosing the peak with the highest amplitude.

Number of Peaks Detected (Burg Method)	Match at the Highest Peak [%]	Match Resulting from Logic Application [%]	Proportion of the Entire Test Dataset [%]
1	100.0	100.0	4.3
2	51.3	91.8 (+40.5)	23.7
3	35.2	90.9 (+55.7)	44.3
4	30.8	86.3 (+55.5)	25.9
>4	8.3	100 (+91.7)	1.8
	40.2	90.5	100.0

Note: Values in parentheses indicate the improvement in percentage points compared to the match at the highest peak.

**Table 2:** The settings of the Infineon XENSIV™ 60 GHz BGT60TR13C radar sensor demo board.

Frame Repetition Time	50 ms
Chirp Repetition Time	0.5 ms
Number of Chirps	1
Start Frequency	58 GHz
End Frequency	63.5 GHz
Sample Rate	3 MHz
Number of Samples	128

**Table 3:** Overall results for the proposed method, presented for all intermediate results, as well as for the MAE and mean relative error (MRE), including the out-of-1σ and out-of-2σ values.

	MRE (%)				MAE (bpm)			
	-	*SD*	Out-of-1-SD	Out-of-2-SD	-	*SD*	Out-of-1-SD	Out-of-2-SD
Stage 1	14.56	11.7	13.43	12.41	11.75	9.22	10.82	9.97
Stage 2	13.24	9.99	12.09	11.05	10.85	8.33	9.91	9.05
Post								
Process	6.88	4.74	5.8	4.88	5.58	4.01	4.69	3.93

## Data Availability

This article includes the original contributions of this research. If you have any questions, please contact the corresponding author.

## Abbreviations

The following abbreviations are used in this manuscript:

ADASs	advanced driver assistance systems
AR	autoregressive
BPM	beats per minute
DMS	driver monitoring system
DWT	discrete wavelet transform
FMCW	frequency-modulated continuous wave
HR	heart rate
HRV	heart rate variability
MAE	mean absolute error
MRE	mean relative error
PSD	power spectral density
RVM	relevance vector machine
SKF	sequential Kalman filter
